# Risk of Optic Pathway Glioma in Neurofibromatosis Type 1: No Evidence of Genotype–Phenotype Correlations in a Large Independent Cohort

**DOI:** 10.3390/cancers11121838

**Published:** 2019-11-21

**Authors:** Giulia Melloni, Marica Eoli, Claudia Cesaretti, Donatella Bianchessi, Maria Cristina Ibba, Silvia Esposito, Giulietta Scuvera, Guido Morcaldi, Roberto Micheli, Elena Piozzi, Sabrina Avignone, Luisa Chiapparini, Chiara Pantaleoni, Federica Natacci, Gaetano Finocchiaro, Veronica Saletti

**Affiliations:** 1 Developmental Neurology Unit, Fondazione IRCCS Istituto Neurologico Carlo Besta, via Celoria 11, 20121 Milan, Italy; giulia.melloni@istituto-besta.it (G.M.); silvia.esposito@istituto-besta.it (S.E.); chiara.pantaleoni@istituto-besta.it (C.P.); 2 Molecular Neuro-Oncology Unit, Fondazione IRCCS Istituto Neurologico Carlo Besta, via Celoria 11, 20121 Milan, Italy; marica.eoli@istituto-besta.it (M.E.); donata.bianchessi@istituto-besta.it (D.B.); mariacristina.ibba@istituto-besta.it (M.C.I.); gaetano.finocchiaro@istituto-besta.it (G.F.); 3 Medical Genetics Unit, Woman-Child-Newborn Department, Fondazione IRCCS Ca’ Granda-Ospedale Maggiore Policlinico, via Francesco Sforza 28, 20122 Milan, Italy; claudia.cesaretti@policlinico.mi.it (C.C.); federica.natacci@policlinico.mi.it (F.N.); 4 Department of Pathophysiology and Transplantation, Pediatric Highly Intensive Care Unit, Università degli Studi di Milano, Fondazione IRCCS Ca’ Granda Ospedale Maggiore Policlinico, via Francesco Sforza 28, 20122 Milan, Italy; giulietta.scuvera@policlinico.mi.it; 5 Paediatric Neurology and Neuromuscular Disorders, University of Genoa and Istituto Giannina Gaslini, Via Gerolamo Gaslini 5, 16147 Genoa, Italy; gmorcaldi@fastwebnet.it; 6 Pediatric Neuropsychiatry, Spedali Civili of Brescia, Piazzale Spedali Civili 1, 25125 Brescia, Italy; microby59@gmail.com; 7 Pediatric Department, ASST Grande Ospedale Metropolitano Niguarda, Piazza Ospedale Maggiore 3, 20162 Milan, Italy; elena.piozzi@ospedaleniguarda.it; 8 Neuroradiology Department, Fondazione IRCCS Ca’ Granda Ospedale Maggiore Policlinico, University of Milan, via Francesco Sforza 28, 20122 Milan, Italy; sabrina.avignone@policlinico.mi.it; 9 Neuroradiology Department, Fondazione IRCCS Istituto Neurologico Carlo Besta, via Celoria 11, 20121 Milan, Italy; luisa.chiapparini@istituto-besta.it

**Keywords:** neurofibromatosis type 1, *NF1* gene, optic pathway glioma, genotype–phenotype correlations

## Abstract

The occurrence of optic pathway gliomas (OPGs) in children with neurofibromatosis type 1 (NF1) still raises many questions regarding screening and surveillance because of the lack of robust prognostic factors. Recent studies of an overall cohort of 381 patients have suggested that the genotype may be the main determinant of the development of OPG, with the risk being higher in patients harbouring *NF1* mutations in the 5’ tertile and the cysteine/serine-rich domain. In an attempt to confirm this hypothesis, we used strict criteria to select a large independent cohort of 309 NF1 patients with defined constitutional *NF1* mutations and appropriate brain images (255 directly enrolled and 54 as a result of a literature search). One hundred and thirty-two patients had OPG and 177 did not. The association of the position (tertiles and functional domains) and type of *NF1* mutation with the development of OPG was analysed using the χ2 test and Fisher’s exact probability test; odds ratios (ORs) with 95% confidence intervals were calculated, and Bonferroni’s correction for multiple comparisons was applied; multiple logistic regression was also used to study genotype–phenotype associations further. Our findings show no significant correlation between the site/type of *NF1* mutation and the risk of OPG, and thus do not support the hypothesis that certain constitutional mutations provide prognostic information in this regard. In addition, we combined our cohort with a previously described cohort of 381 patients for a total of 690 patients and statistically re-analysed the results. The re-analysis confirmed that there were no correlations between the site (tertile and domain) and the risk of OPG, thus further strengthening our conclusions.

## 1. Introduction

Neurofibromatosis type 1 (NF1) is a multi-system, tumour-prone disorder that is diagnosed on the basis of established clinical criteria [1,2]. It is one of the most frequently inherited genetic conditions (its worldwide prevalence is about 1/3000 people), and it is characterised by complete and age-related penetrance and highly variable expression [3,4,5]. It is caused by constitutional dominant loss-of-function intragenic mutations or the deletion of the *NF1* gene (OMIM 613113) located on the long arm of human chromosome 17. With its 61 exons, four of which are alternatively spliced, *NF1* is one of the largest genes and has one of the highest mutation rates in the human genome [6,7].

The gene encodes for neurofibromin, a ubiquitous 2818 amino acid protein with various domains, the most widely known of which is the GTPase-activating protein (GAP)-related domain (GRD), which exerts tumour suppressing activity by down-regulating the Ras signalling pathway [8].

Although less well-characterised, the other *NF1* domains that have been described are the cysteine/serine-rich domain (CSRD), the tubulin-binding domain (TBD), the Sec14 homology-like (Sec14) domain, the pleckstrin homology-like (PH) domain, the HEAT-like repeat regions (HLR), the C-terminal domain (CTD), the nuclear localisation signal region (NLS), and the syndecan-2 binding region (SBR) [8].

The enormous number of reported pathogenic *NF1* variations consist of intragenic *NF1* mutations, which are found in about 90% of NF1 patients, and large 17q11.2 deletions encompassing the entire *NF1* gene and a number of flanking genes (the *NF1* microdeletion), which are found in 5%–10% [9]. Point mutations are observed in all exons and are mostly nulling or protein-truncating mutations, while a minority (9.4%–15%) are missense mutations [10,11,12].

The reported detection rates range from 60% to 97% depending on the technique used and the source of the tissues. Advances in genetic investigation techniques have allowed a molecular diagnosis to be made in the majority of cases using multiple gene DNA and RNA screening approaches: Sanger sequencing, next-generation sequencing (NGS), multiplex ligation-dependent probe amplification (MLPA), and array comparative genomic hybridisation CGH [9,10,11].

NF1 patients may present a wide range of multi-system complications and are prone to developing benign and malignant tumours of the central and peripheral nervous systems, as well as systemic malignancies [13]. They consequently need periodic monitoring in order to minimise the risk of serious medical complications [14]. About 15%–20% of patients develop low-grade glial tumours, 80% of which involve the optic pathway [15].

Optic pathway glioma (OPG), a predominantly World Health Organisation (WHO) grade I astrocytoma, is one of the diagnostic criteria for NF1 and the most frequently identified brain tumour in NF1 children [1,15]. In NF1 children aged <7 years, it can arise anywhere along the optic pathway from the retro-orbital optic nerve to the post-chiasmatic optic traits and radiations. It is usually infiltrative, has a low proliferative index, and there is no defined cystic component. Although its course is more indolent and favourable than sporadic optic pathway glioma, and it occasionally regresses spontaneously [16,17,18,19], up to half of the patients develop clinical symptoms, such as reduced visual acuity, pale papillae of the optic nerve, strabismus, proptosis, and endocrinological abnormalities, particularly precocious puberty [20].

OPG in NF1 patients still raises many questions regarding screening and surveillance [21]. Contrast-enhanced brain magnetic resonance imaging (MRI) is the standard imaging technique used to identify and monitor OPG [2,22], but it is very costly and its use for screening purposes is controversial. The Optic Pathway Task Force has recommended against it because radiological and clinical findings do not match sufficiently, asymptomatic OPGs do not require treatment, and anesthesia is a risk for small children. However, an early OPG diagnosis in young children may improve the outcome [15,21,23].

There is a general consensus regarding the usefulness of an ophthalmological evaluation at least once a year up to 10 years of age, but visual acuity measurements are not always reliable in small children [15,24].

In this context, the challenge is to predict which children may develop OPG and which OPG may reduce visual acuity and/or lead to symptoms and signs.

Tumour localisation in the optic chiasm and/or posterior optic tracts, an early age at the time of tumour onset, and being a female all seem to lead to a worse prognosis, but the evidence is not sufficient to propose different surveillance strategies [15,24].

Over the last few years, many authors have focused on genotype–phenotype correlations in patients with NF1, and the findings of some studies suggest that the genotype may be the main determinant of the development of OPG in NF1 children.

Patients with *NF1* gene mutations in the 5’ tertile (exon 1–21) and the CSRD (residues 543–909) seem to be at a higher risk of developing OPG [25,26,27,28,29], whereas patients with mutations in the HLR (residues 1825–2428) located in the 3’ tertile (exon 39–57) seem to be at lower risk [28]. In addition, OPG seems to be more strongly associated with patients with 5’-end truncating or nonsense mutations [27].

The aim of this collaborative retrospective study was to confirm these correlations by gathering information concerning a large independent cohort of unrelated NF1 patients with defined constitutional *NF1* mutations who have undergone brain MRI.

Identifying an association between the risk of developing OPG and specific *NF1* mutations is an attractive prospect as it would be very helpful in genetic counselling, as well as in guiding screening and surveillance during childhood. In the long term, it may also be useful in further clarifying the pathogenesis of OPGs and developing targeted treatments.

## 2. Materials and Methods

### 2.1. Study Subjects

The study involved patients from four regional NF1 referral centres: Fondazione IRCCS Istituto Neurologico Carlo Besta and Fondazione IRCCS Ca’ Granda-Ospedale Maggiore Policlinico in Milan, Istituto Giannina Gaslini in Genoa, and Spedali Civili in Brescia.

The study inclusion criteria were as follows:

- a clinical diagnosis of NF1 based on the 1988 NIH diagnostic criteria [1];
- a defined constitutional *NF1* mutation;
- brain MRI scans available in the institutional picture archiving and communication system (PACS).

We excluded the following:

- the youngest patients in familial cases in order to include patients with a more expressed phenotype and to avoid any confounding effects due to shared polymorphisms in possible modifying genes in family members;
- patients in whom the entire *NF1* gene and flanking genes were deleted (microdeletion) because it is not possible to study the correlation between the localisation of the mutation and phenotype in such patients;
- patients harbouring more than one variant of the *NF1* gene because the role of each variant needs to be tested separately;
- patients with increased optic nerve tortuosity and nerve or sheath thickening without any clear signs of OPG because of the uncertain significance of these characteristics in terms of the subsequent development of OPG [30]; and
- patients aged <10 years without an MRI detected OPG because they are still at risk of developing OPG.

The patients were selected using the clinical data in their electronic medical records. The patients or their parents signed an informed consent form before MRI and genetic testing were carried out, and gave their consent to the use of the anonymous results for retrospective research studies. The investigations were carried out in accordance with the principles laid down in the 2013 revision of the Declaration of Helsinki. This retrospective study is part of an NF1 research line approved by the Fondazione IRCCS Istituto Neurologico Carlo Besta Scientific Board and, in accordance with Italian regulations, does not require specific ethical approval because it only uses anonymous data collected during routine patient care.

The indications for brain MRI were neurological and/or visual signs and symptoms, baseline screening at the time of the first evaluation, and radiological surveillance of OPG or other brain lesions.

The diagnosis of OPG in patients with NF1 is currently based on the pathognomonic characteristics of brain MRI; an OPG biopsy is not usually used because of the high risk of vision loss [31].

The MRI examinations were performed using a 1.5 T or 3 T machine, and concentrated on the chiasmatic and orbital regions (differences in MRI resolution do not interfere with the detection of OPG). Two senior neuroradiologists (L.C. and S.A.) with more than 20 years’ experience reviewed the neuroimages and confirmed the diagnosis of OPG on the basis of the pathognomonic characteristics of the brain MRI scans. Although there is no universally accepted radiological definition of NF1-associated OPG, the standard is an MRI scan of the brain and orbits using thin slices through the optic nerves and chiasm, including T1-weighted sequences with and without gadolinium, and T2-weighted sequences. As contrast enhancement may be heterogeneous, T2-weighted sequences often define the tumour borders more accurately [31,32]. OPGs may show enlargement of the optic nerve, chiasm, optic tracts, and optic radiations. Gliomas of the optic nerve usually have a tubular/fusiform appearance with an often downward kink, whereas chiasmal gliomas can appear as an enlargement of the chiasm or a suprasellar mass. The radiographical interpretation of OPGs can be complicated by T2-hyperintense lesions (which are frequently detected in the brain of NF1 children) overlapping the borders of the tumour. In addition, OPGs are extra-axial and intra-dural and remain within the nerve sheath, which means they may resemble cerebrospinal fluid in T2-weighted sequences (the “pseudo-CSF” sign) [24,31,32]. Tortuous optic nerves and optic nerve or sheath enlargements are quite frequently observed in MRI scans of NF1 children, but their significance in terms of the subsequent development of OPG is debated [30]. In order to avoid missing or over-diagnosing OPGs, any doubtful cases without clear signs of an OPG (i.e., nerve tortuosity, suspected nerve, or sheath thickening) were excluded.

The patients were divided into two groups: NF1 patients of any age with an MRI confirmed diagnosis of OPG were included in the NF1 OPG group, whereas NF1 patients aged ≥10 years without any optic pathway tumour, as confirmed by MRI, were included in the NF1 non-OPG group (patients who have not developed an OPG by the age of 10 years are extremely unlikely to do so in later life) [21]. A total of 255 patients were selected and included in the study: 92 with OPG and 163 without. The MRI data and ages reported herein are those of the latest clinical evaluation by the referring physicians co-authoring this paper.

### 2.2. Literature Review

We systematically reviewed the recent scientific literature with the aim of increasing the number of cases to a minimum of 307 patients, because Anastasaki et al. have calculated that this number is needed to detect an effect size of 0.16 with 80% power using a chi-squared test to detect differences in proportions with an alpha level of 0.05 [27].

We looked for NF1 patients who had not been described in previous studies of genotype–phenotype correlations in relation to the development of OPG [27,28,29]. The studies were identified by searching the Pubmed (2017–present) and Google scholar (2017–present) medical databases using the following terms: NF1, neurofibromatosis, and genotype–phenotype or genotype or phenotype. 

We only selected patients fulfilling the inclusion and exclusion criteria described above. In particular, we excluded all reports without enough information to allow us to assign patients to the OPG or non-OPG group. In order to be included in the non-OPG group, the patients had to have undergone negative MRI after the age of 10 years. The OPG group only included patients with a reported neuroradiological diagnosis of OPG; we did not have access to the radiological images, but assumed that the reported data were correct. We also excluded known familial cases (we checked whether there was consanguineity in patients sharing the same mutation). The only patients with the same mutation were patient number 144 in the series described by Tsipi et al. [33] and patient number 10 in the series described by Ulusal et al. [34] and, as these patients belonged to different cohorts and their mutation is known to be frequent, there is little likelihood of consanguineity. The two patients harbouring the same *NF1* mutation and retrieved from the paper by Trevisson et al. belonged to unrelated families [35].

The literature review allowed us to select 54 patients; there were more patients in the OPG group (40) than in the non-OPG group (14) [33,34,35,36,37,38,39,40,41].

### 2.3. Molecular Testing and Analysis of NF1 Mutations

Blood samples from 238 of the 255 patients attending the study centres were originally sent to the laboratory of the Molecular Neuro-oncology Unit of Fondazione IRCCS Istituto Neurologico Carlo Besta for molecular *NF1* genetic testing in order to establish or confirm the diagnosis of NF1.

Constitutional intragenic *NF1* mutations were determined by means of standard techniques using gDNA and, more recently, cDNA; that is, Sanger sequencing and NGS to test for point mutations, and MLPA to test for intragenic deletion/duplication [11,38].

Genomic DNA was obtained from blood EDTA samples, and RNA samples were collected in Tempus blood RNA tubes and reverse-transcribed into cDNA. NGS was carried out using the Ion Sequencing Kit v2.0. Sanger sequencing was used for the oldest samples, for the positive amplicons and the cDNA of negative amplicons obtained by means of NGS Ion Torrent sequencing. MLPA was carried out using *NF1* MLPA salsa P081 and P082. The variants were defined in accordance with the reference sequence NM_000267.3 and human reference genome hg19.

When in vitro studies were not available, the effect of the identified mutations on genes and proteins were predicted by querying various prediction sites, including Mutation Taster (http://www.mutationtaster.org), and databases such as the National Center for Biotechnology Information (NCBI) Single Nucleotide Polymorphism database (dbSNP) and the 1000 Genomes Project (TGP) database: disease variants came from dbSNP (ClinVar) and from the Human Genome Mutation Database (HGMD) PolyPhen-2 (https://genetics.bwh.harvard.edu./pph2/). The possible effects on mRNA (canonical and non-canonical splicing mutations) were evaluated using neural network Splice site Prediction (http://www.fruitfly.org/seq_tools/splice.html) [42], the Human Splicing Finder (HSF; http:www.umd.be/HSF/) [43], and the ESE Finder (http://rulai.cshl.edu/cgibin/tools/ESE3/esefinder.cgi?process=home) [44].

The mutations were classified as nonsense (NS), frameshift (FS), missense (MS), inframe (ID), or splicing (SS) mutations; or large deletion (LD) consisting of at least an entire exon. Care was taken when interpreting the effects of the mutations on proteins as some putative MS mutations may also cause splicing abnormalities [10]; the exonic variations with effects on splicing were included in the splicing group. The novel mutations identified in this study were deposited with the LOVD database (http://www.LOVD.nl/NF1), and are described in accordance with HGSV recommendations.

**Figure 1:**
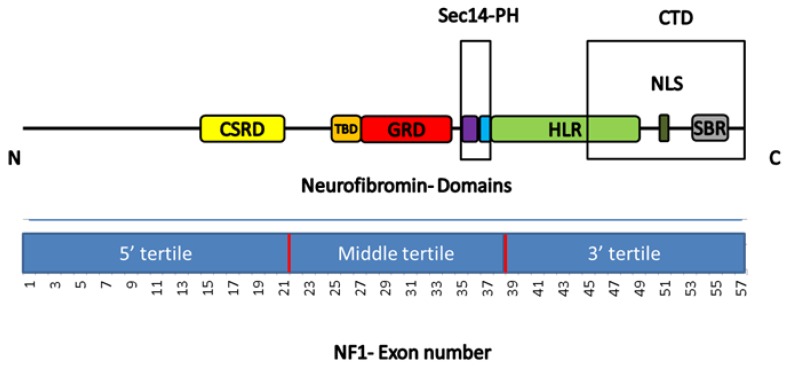
Schematic representation of the tertiles of the neurofibromatosis type 1 (*NF1*) gene and the domains of neurofibromin. CSRD, cysteine/serine-rich domain; TBD, tubulin-binding domain; GRD, GTPase activating protein-related domain; PH, pleckstrin homology-like domain; Sec-14, Sec14-like domain; HLR, HEAT-like repeat regions; NLS, nuclear localisation signal region; CTD, C-terminal domain; SBR, syndecan-binding region.

On the basis of the criteria described by Sharif [25], the mutations were attributed to tertiles: the 5’ tertile spanning exons 1–21, the middle tertile spanning exons 22–38, and the 3’ tertile spanning exons 39–57. The mutations were also mapped to a specific domain as indicated by Xu [28], with residues 543–909 belonging to the cysteine/serine-rich domain (CSRD), residues 1095–1197 to the tubulin-binding domain (TBD), residues 1198–1530 to the GTPase activating protein-related domain (GRD), residues 1560–1705 to the Sec14-like domain (Sec14), residues 1716–1816 to the pleckstrin homology-like domain (PH), residues 1825–2428 to the HEAT-like repeat regions (HLR), residues 2260–2817 to the C-terminal domain (CTD), residues 2534–2550 to the nuclear localisation signal region (NLS), and residues 2619–2719 to the syndecan-binding region (SBR) [45,46,47]. The *NF1* tertiles and neurofibromin domains are schematically shown in Figure 1.

### 2.4. Statistical Analysis

The χ2 test and Fisher’s exact probability test were used to compare the frequencies of the independent variables (the locations of mutations by tertile and domain, and the type of mutations) between the NF1 OPG and the NF1 non-OPG group. Odds ratios (ORs) and their 95% confidence intervals (CIs) were calculated. Genotype–phenotype associations were studied using multiple logistic regression. Bonferroni’s method was used to correct *p*-values for multiple testing. *p*-values of <0.05 were considered statistically significant. The descriptive, frequency, and comparative statistical analyses were carried out using SPSS 22.0 software.

## 3. Results

A total of 309 NF1 patients were selected on the basis of the inclusion and exclusion criteria and included in the study; 255 were recruited from the authors’ institutions (92 with OPG and 163 without OPG) and 54 were retrieved from the recent literature (40 with OPG and 14 without OPG) [33,34,35,36,37,38,39,40,41]. One hundred and thirty-two patients were included in the NF1 OPG group (61 females, 57 males; the sex of 14 patients retrieved from the literature was not reported) and 177 in the NF1 non-OPG group (91 females, 76 males; 10 unknown). Table 1 shows their clinical details (age, sex, OPG diagnosis); the molecular details (DNA, RNA, protein change); and the classification of the variants by type, tertile, and domain.

### 3.1. Distribution of NF1 Mutations by Tertile and the Risk of Developing OPG

**Figure 2:**
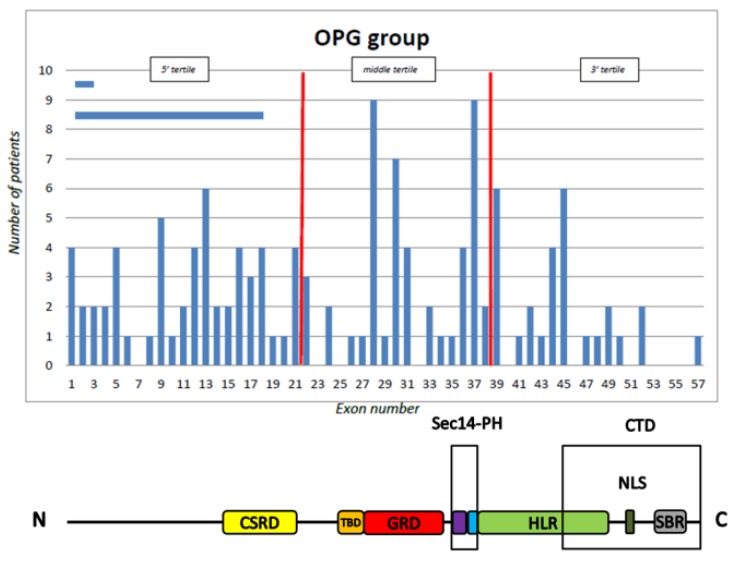
Distribution of *NFI* gene mutations by tertile and domain in the optic pathway glioma (OPG) group. The horizontal bars indicate two OPG patients with a large deletion involving more than one exon. The vertical red lines indicate the limits of the tertiles.

**Figure 3:**
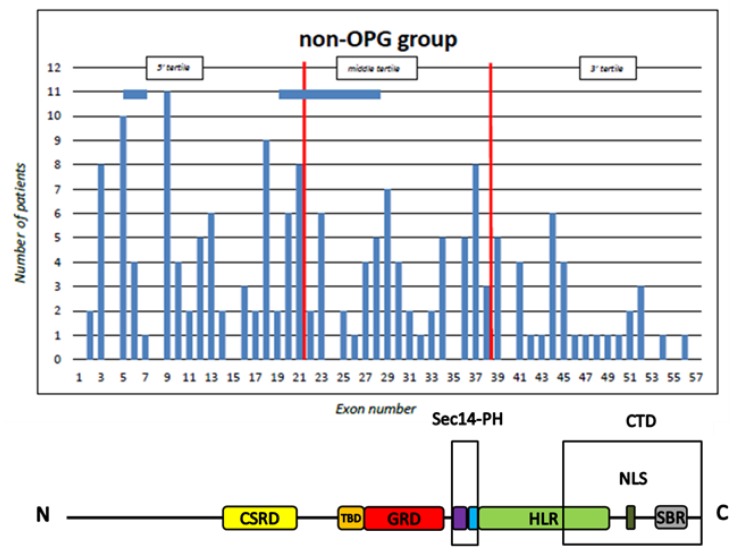
Distribution of *NF1* gene mutations by tertile and domain in the non-OPG group. The horizontal bars indicate two OPG patients with a large deletion involving more than one exon. The vertical red lines indicate the limits of the tertiles.

The total number of patients harbouring mutations located in the 5’, middle, and 3’ tertile was 144 (46.60%, CI = 41.12%–52.17%), 104 (33.66%, CI = 28.62%–39.10%), and 61 (19.74%, CI = 15.69%–24.54%), respectively. The distribution of mutations was 57 (43.2%) in the 5’ tertile, 47 (35.6%) in the middle tertile, and 28 (21.2%) in the 3’ tertile in the NF1 OPG group, and 87 (49.2%), 57 (32.2%), and 33 (18.6%), respectively, in the NF1 non-OPG group (Figure 2 and Figure 3).

There were no statistically significant differences between the two groups; in particular, the patients with OPG were not more likely to harbour 5’ tertile mutations than those without OPG (43.6 vs. 49.2, *p* = 0.29) (Table 2).

### 3.2. Distribution of NF1 Mutations by Domain and the Risk of Developing OPG

The analysis was extended to all of the *NF1* domains in order to evaluate whether the risk of developing OPG was associated with mutations in the CSRD and HLR, as previously reported. There were no statistically significant differences between the OPG and the non-OPG group. In particular, there were 20 mutations (15.2%) in the CSRD in the OPG group, and 30 (16.9%) in the non-OPG group (*p* = 0.67); and 25 mutations (18.9%) in the HLR domain in the OPG group, and 29 (16.4%) in the non-OPG group (*p* = 0.55) (Table 3).

### 3.3. Spectrum of Mutation Types and the Risk of Developing OPG

The number of patients with frameshift, nonsense, splicing, missense, and inframe mutations and large deletions was 101 (32.7%), 85 (27.5%), 76 (24.6%), 33 (10.7%), 6 (1.9%), and 8 (2.6%), respectively.

The proportion of nonsense mutations was higher in the OPG group than in the non-OPG group (OR 1.77; CI = 1.07–2.93). However, when the *p*-value was adjusted for multiple comparisons, the difference was not statistically significant (*p* = 0.15) (Table 4).

## 4. Discussion

The phenotypic expression of NF1 varies widely from mild cutaneous manifestations to serious complications, and is largely unpredictable because of the lack of robust prognostic risk factors. This unpredictability is one of the most difficult aspects of disease management for both patients and clinicians as it makes the genetic counselling and screening and surveillance of NF1 patients extremely challenging [13,14,48].

In this context, any suggestion of a possible genotype–phenotype correlation arouses considerable interest as it might aid clinical care by offering an opportunity to better inform patients about what to expect in the future, and allowing personalised screening and monitoring based on changes in the *NF1* gene.

NF1 genotype–phenotype correlation studies began to be developed after the identification of the *NF1* gene in 1990 [49,50], but they were limited by the difficulty of detecting *NF1* mutations until the recent development and spread of new genetic diagnostic investigational techniques gave them renewed impulse [51].

However, identifying specific genotype–phenotype correlations is still very challenging because of the variability of the clinical presentation of the disease, the age-dependence of most of its features, the wide range of *NF1* gene variants (often considered private variations), and the timing and number of second hits in specific cells [52]. Furthermore, given the phenotypic variability even among patients with familial NF1 who share the same germline mutation, it has been suggested that there are likely to be other influences such as modifier genes, as well as epigenetic and environmental factors, although their contribution to determining the phenotype is still unknown and may vary depending on disease traits [25].

Despite all of these limitations, at least four genotype–phenotype correlations in NF1 have recently emerged, and others are still being studied.

Firstly, patients with large deletions of the *NF1* gene region and flanking genes (*NF1* microdeletions) tend to present a more severe phenotype in terms of cognitive impairment and/or learning disabilities, facial dysmorphisms and cardiovascular malformations, and are at increased risk of malignant peripheral nerve sheath tumours (MPNSTs) [53].

Secondly, two specific intragenic mutations (a 3-bp in-frame deletion *NF1* c.2970_2972 del p.Met992del, and missense mutations affecting p.Arg1809) have been associated with distinct, but partially concordant mild clinical phenotypes, with the first being characterised by the absence of cutaneous neurofibromas and other serious complications [51,54], and the second by the absence of cutaneous or plexiform neurofibromas with Noonan–like features [55,56].

Finally, it has recently been found that patients with missense mutations involving one of the codons 844–848 within the CSRD have a more severe phenotype and are at a higher risk of developing MPNSTs, OPGs, and malignant neoplasms [52].

In addition to these four genotype–phenotype correlations, the findings of recent studies suggest that the type and the position of *NF1* mutations may be the main determinant of the risk of developing spinal neurofibromas (patients more likely to have MS or SS mutations) [57], pulmonary stenosis (a significantly higher prevalence of non-truncating *NF1* mutations) [58], breast cancers (a higher proportion of both nonsense and missense mutations) [12], and OPGs.

In 2011, Sharif et al. were the first to find a trend toward the clustering of pathogenic changes in the 5′ tertile (exons 1–21) of the *NF1* gene in 29 NF1-OPG patients from the United Kingdom [25], and the same trend was found in another cohort of 20 NF1-OPG patients with 12 different mutations described by Ars [59]. In order to determine whether the risk of developing OPG was associated with mutation location, Sharif et al. combined their finding with those of Ars and a larger series of Castle et al. [60] to give a total of 125 NF1 patients (predominantly from the United Kingdom), 36 of whom had OPGs. Statistical analysis showed a significant distribution of mutations in the 5’ tertile of the *NF1* gene in NF1 patients with OPGs when compared with NF1 patients without OPGs.

The clustering of mutations in the 5’ tertile of NF1 gene in patients with OPG was subsequently supported by Bolcekova et al., who observed a similar *NF1* mutation pattern in their series of 25 Slovakian NF1-OPG patients in comparison with 27 NF1 controls [26]. They found that 71% of the mutations in OPG patients were located in the first tertile as against 29% in the non-OPG group. They believed that the CSRD, which spans exons 11–17 in the first tertile of the gene, may be as important a functional domain as the RAS-GAP domain, and may play an important role in the development of OPG.

These associations were not confirmed by a subsequent study by Hutter et al. [61], who used stricter inclusion criteria to select 77 NF1 patients from Germany and Canada (thus reducing regional genetic differences), and found a point mutation in 37 OPG patients and 32 controls. They observed a greater proportion of mutations in the 5’ region in both the OPG (17/37, 46%) and non-OPG patients (15/32, 47%). In order to avoid misinterpreting data from small cohorts, they combined their series with that of Bolcekova et al., thus increasing the cohort to 129 NF1 patients (66 OPG and 63 non-OPG), but found no significant correlation between mutations in the 5’ tertile of *NF1* and the development of OPG.

In 2017, Anastasaki et al. analysed 37 NF1 patients at Washington University (14 with OPG and 13 without) in order to ascertain the relationship between the type and the location of the germline *NF1* mutation and the presence of OPG, and did not find any statistically significant correlation [27]. However, power calculations revealed that a sample size of 307 patients is required to determine the predictive value of the position or type of *NF1* gene mutations, and so they combined their data set with those of the four previous studies by Ars, Sharif, Bolcekova, and Hutter to reach a sample size of 310 patients. It was found that children with OPG were more likely to harbour 5’ gene mutations. In addition, the association was stronger in subjects with 5’ tertile truncating or nonsense mutations, thus suggesting that the *NF1* mutation may be a predictive factor [27].

However, using stricter inclusion and exclusion criteria, Xu et al. found no significant statistical association between mutations clustering in the 5’ tertile and the risk of developing OPG when they analysed 215 NF1 patients (5 from Sun Yatsen University in China and 210 selected from the literature) [28]. Nevertheless, it is interesting to note that patients with mutations involving the cysteine/serine-rich domain of *NF1* were at a higher risk of developing OPG than those with mutations in other regions, whereas those with mutations in the HEAT-like repeat region were at a lower risk. As different mutation types have different effects on protein structure and function, the authors also compared the distribution of the types in the two regions, and found that nonsense mutations were more frequent in the CSRD in the OPG group and splicing site mutations were more frequent in the HLR in the non-OPG group, but these between-region differences were not significant [28].

As recently as 2019, Anastasaki et al. [29] re-examined their previously reported data concerning patients from their institution and retrieved from the literature (a total of 310 patients) and the dataset of Xu et al. (215 patients, 144 of whom were the same as those in the dataset of Anastasaki et al.), and found that mutation clustering in the 5’ tertile was significantly different between the patients with and without OPG when their dataset was considered alone (310 patients) or combined with that of Xu (making a total of 381 patients), but not when Xu’s dataset was considered alone. In addition, an analysis of their own cohort of 310 patients confirmed the findings of Xu et al., that mutations in the CSRD were significantly positively associated with OPG, whereas mutations in the HLR were negatively associated with OPG, and this difference persisted when the combined dataset of 381 patients (127 with OPG and 254 without) was analysed.

This succession of papers with partially concordant findings about a correlation between OPG and *NF1* mutations located in the first tertile of the gene or in the CSRD prompted us to contribute to the current debate concerning NF1 genotypes and the risk of developing OPG by validating these findings in a large independent series of patients.

Unfortunately, and unexpectedly, our data do not confirm the association reported by Xu et al. and Anastasaki et al., or the original observations of Sharif et al., as we did not find any correlation between the development of OPG and the site (tertile and domain) or type of mutation [25,27,28,29].

The difference may be because of what we consider to be the most important limitation of the previous studies: patient enrolment. The studies by Sharif, Hutter, and Bolcekova were limited by their small sample sizes; in addition, some included familial cases and children under 10 years of age in the non-OPG group (even though the former can clearly carry the same modifier genes and the latter are still at risk of developing an OPG), and patients with whole gene deletions [25,26]. Furthermore, with the aim of reaching a large series with statistical power, Xu and Anastasaki progressively added a limited number of patients to the original cohorts of Sharif, Hutter, and Bolcekova [27,28,29] and, although they filtered the patients retrieved from the literature using stricter inclusion and exclusion criteria before the statistical re-analyses, a bias was generated because the same patients were repeatedly analysed in the various studies.

The strengths of the present study include the fact that it involved a large and completely independent sample of NF1 patients selected on the basis of strict inclusion and exclusion criteria and did not include family cases, patients harbouring a microdeletion, or patients aged <10 years in the non-OPG group. In addition, our series mainly consists of patients who were genetically analysed by a single laboratory and selected using precise radiological criteria for a diagnosis of OPG. Cases with increased optic nerve tortuosity and nerve or sheath thickening were excluded because it is unclear whether these characteristics are significant in terms of the subsequent development of OPG [30].

In order to achieve the statistical power described by Anastasaki et al., we searched the literature for patients meeting the same inclusion/exclusion criteria as those used to select our institutional cohort. We decided not to select any patients previously included in the studies of Anastasaki, Xu, and the others mentioned above in order to ensure a completely independent cohort. 

However, to provide additional data, we combined our cohort with the most recent cohort described by Anastasaki [29] to create a large sample of 690 patients, and performed the same statistical analysis. The results confirmed the absence of a correlation between the position of the NF1 mutation (by tertile and functional domain) and the risk of OPG, thus further strengthening our conclusions (Tables S1 and S2).

It is worth noting that some of the patients analysed in our study (those in our institutional cohort and those retrieved from the literature) had different ethnic origins. We think that is another strength of the study as it limits the potential confounding effect of polymorphisms shared by the same ethnic group. 

However, our study also has some limitations. The first is that the 30.3% of our OPG patients were retrieved from the literature and we cannot be sure that the same radiological criteria were used in their diagnosis.

In addition, when selecting the patients in the non-OPG group, unlike Hutter et al. [61], we did not consider non-optic gliomas among the exclusion criteria. Hutter et al. probably assumed that gliomas have the same biological basis in NF1 patients regardless of their location, but we believe that the biological basis of optic and non-optic gliomas is probably different because they occur at different patient ages and are histologically different (non-optic gliomas are more aggressive and OPGs are more stable). Moreover, adult patients are at risk of developing non-optic gliomas and, if we had used the same exclusion criterion as Hutter et al., we would only have been able to include patients at low risk; that is, very old patients.

Lastly, it would be very interesting to examine the correlations between site/type of *NF1* mutations and other factors such as tumour size; position in the optic pathway; and, above all, symptomatology, because this could help to clarify why some OPGs behave aggressively and others have a stable and benign course over time; however, this would require a much larger sample of NF1 patients with OPG.

## 5. Conclusions

Knowing the type and location of *NF1* mutations does not give patients or clinicians any valuable prognostic information regarding the development of OPG.

In order to identify other genotype–phenotype correlations, it is necessary to select extremely large and homogeneous groups of patients on the basis of their genotype or phenotype. Previous examples of recognised genotype–phenotype correlations in NF1 have involved patients with a single recurrent mutation or the deletion of the entire *NF1* gene, who make much more homogeneous groups.

Our findings seem to support the hypothesis that other modifying influences such as modifier genes, as well as epigenetic and environmental factors, are likely to be involved in determining the NF1 phenotype.

## Figures and Tables

**Table 1:** Clinical data, molecular details, mutation type, and mutation location by tertile and domain.

ID Code	Age	Sex	OPG	DNA Change	RNA Change	Protein Change	Type	Tertile	Domain
162	60	f	yes	c.1-?_60+? del	r.(?)	p.(?)	LD	1	nd
6	30	f	yes	c.21_22delGG	r.(?)	p.Glu8Metfs*29	FS	1	nd
112	15	f	yes	c.60+1G>A	r.(?)	p.(?)	SS	1	nd
Calì et al. 1 [38]	29	f	yes	c.61-2A>C	r.(?)	p.(?)	SS	1	nd
8	12	f	yes	c.61-?_204+?del	r.61_204del	p.Leu21_Met68del	LD	1	nd
24	31	f	no	c.61-?_288+?del	r.61_288del	p.Leu21_Gly96del	LD	1	nd
139	6	f	yes	c.61-?_288+?del	r.61_288del	p.Leu21_Gly96del	LD	1	nd
Bonatti et al. 1 [37]	na	m	yes	c.61-?_2325+?del	r.(?)	p.Leu21_Glu775del	LD	1	CSRD
Tsipi et al. 38 [33]	55	na	no	c.86_87delAC	r.(?)	p.His31Tyrfs*6	FS	1	nd
136	35	f	no	c.99A>G	r.100_204del	p.Val34_Met68del	SS	1	nd
177	11	f	yes	c.185dupT	r.(?)	p.Leu62Phefs*5	FS	1	nd
76	42	f	no	c.205_288del	r.205_288del	p.Arg69_Gly96del	LD	1	nd
153	21	m	no	c.236T>G	r.(?)	p.Leu79*	NS	1	nd
46	36	f	yes	c.247C>T	r.(?)	p.Gln83*	NS	1	nd
65	9	f	yes	c.247delCinsGAGA	r.(?)	p.Gln83delinsGluLys	ID	1	nd
30	53	m	no	c.252delG	r.(?)	p.Ile85fs*18	FS	1	nd
127	59	m	no	c.259_264delTTGGATinsAA	r.259_264deluuggauinsaa	p.Leu87Lysfs*15	FS	1	nd
109	71	f	no	c.288+1delG	r.288_288del	p.Gln97Asnfs*6	SS	1	nd
250	34	m	no	c.288+5G>A	r.205_288del	p.Arg69_Gly96del	SS	1	nd
256	26	f	no	c.288+5G>C	r.(?)	p.(?)	SS	1	nd
244	33	m	no	c.288+1138C>T	r.288_289 ins 288+1019_288+1136ins118	p.Gly96_Glu 97ins39aa+fs *10	SS	1	nd
Tsipi et al. 114 [33]	7	na	yes	c.350_351insT	r.(?)	p.Cys118Leufs*9	FS	1	nd
79	35	f	yes	c.479G>T	r.289_479del	p.Gln97Valfs*13	SS	1	nd
31	75	f	no	c.484C>T	r.(?)	p.Gln162*	NS	1	nd
7	44	m	no	c.493delA	r.(?)	p.Thr165Leufs*13	FS	1	nd
131	50	f	no	c.495_498delTGTT	r.495_498del	p.Thr166fs*11	FS	1	nd
68	28	f	yes	c.495_498delTGTT	r.495_498del	p.Thr166fs*11	FS	1	nd
61	39	f	no	c.499_502delTGTT	r.(?)	p.Cys167Glnfs*10	FS	1	nd
183	26	m	no	c.499_502delTGTT	r.(?)	p.Cys167Glnfs*10	FS	1	nd
Terzi et al. 23 [40]	na	na	yes	c.499_502delTGTT	r.(?)	p.Cys167Glnfs*10	FS	1	nd
Tsipi et al. 90 [33]	15	na	yes	c.501T>A	r.(?)	p.Cys167*	NS	1	nd
171	29	m	no	c.539T>G	r.539u>g	p.Leu180*	NS	1	nd
44	57	f	no	c.574C>T	r.574c>u	p.Arg192*	NS	1	nd
57	44	f	no	c.574C>T	r.574c>u	p.Arg192*	NS	1	nd
160	17	f	yes	c.574C>T	r.574c>u	p.Arg192*	NS	1	nd
188	56	f	no	c.586+1G>T	r.(?)	p.(?)	SS	1	nd
180	44	m	no	c.586+4dupA	r.(?)	p.(?)	SS	1	nd
193	46	f	no	c.647_649delTGG	r.(?)	p.Leu216_Glu217delinsGln	ID	1	nd
175	27	f	no	c.615_616delGAinsAT	r.587_654del	p.Glu196Glyfs*12	SS	1	nd
85	42	m	no	c.649delG	r.(?)	p.Glu217Lysfs*8	FS	1	nd
144	38	f	no	c.652_653delAAinsG	r.(?)	p.Lys218Glyfs*7	FS	1	nd
Tsipi et al. 156 [33]	4	na	yes	c.653_653insA	r.(?)	p.Lys218Argfs*7	FS	1	nd
215	11	m	no	c.681T>G	r.(?)	p.Tyr227*	NS	1	nd
1	25	m	yes	c.801delG	r.(?)	p.Trp267Cysfs*14	FS	1	nd
253	59	f	no	c.910C>T	r.(?)	p.Arg304*	NS	1	nd
118	30	f	no	c.910C>T	r.(?)	p.Arg304*	NS	1	nd
129	38	m	yes	c.945_946delGCinsAA	r.889_1062del	p.Lys297_Lys354del	SS	1	nd
228	15	m	no	c.952_953delGA	r.(?)	p.Glu318fs*11	FS	1	nd
135	15	m	yes	c.952_953delGA	r.(?)	p.Glu318fs*11	FS	1	nd
Tsipi et al. 102 [33]	44	na	no	c.968C>A	r.(?)	p.Ala323Asp	MS	1	nd
224	15	m	no	c.980T>C	r.980u>c	p.Leu327Pro	MS	1	nd
120	15	m	yes	c.980T>C	r.980u>c	p.Leu327Pro	MS	1	nd
212	15	f	no	c.998_999insA	r.998_999insa	p.Tyr333*	FS	1	nd
199	4	f	yes	c.1007G>A	r.(?)	p.Trp336*	NS	1	nd
35	35	f	no	c.1019_1020delCT	r.(?)	p.Ser340Cysfs*12	FS	1	nd
106	20	m	no	c.1021_1022delGT	r.(?)	p.Val341Hisfs*11	FS	1	nd
Tsipi et al. 78 [33]	15	na	no	c.1022_1023insGA	r.(?)	p.Ile342Thrfs*35	FS	1	nd
166	na	f	no	c.1062G>T	r.889_1062del	p.Lys297_Lys354del	SS	1	nd
105	48	f	yes	c.1062+113A>G	r.1062_1063ins113	p.Asn355Valfs*12	SS	1	nd
170	18	m	no	c.1063-2A>C	r.(?)	p.(?)	SS	1	nd
84	44	f	no	c.1122_1125delTCTA	r.1122_1125del	p.Asp374Glufs*2	FS	1	nd
74	36	f	no	c.1140delT	r.(?)	p.Val381Phefs*6	FS	1	nd
Bonatti et al. 15 [37]	na	m	yes	c.1144delT	r.(?)	p.Ser382Leufs*5	FS	1	nd
Tsipi et al. 126 [33]	28	na	no	c.1182_1183insT	r.(?)	p.Lys395*	FS	1	nd
59	45	f	no	c.1186-1G>C	r.1186_1200del	p.Ile396_Gln400del	SS	1	nd
51	9	m	yes	c.1246C>T	r.1246c>u	p.Arg416*	NS	1	nd
208	10	f	no	c.1246C>T	r.1246c>u	p.Arg416*	NS	1	nd
238	20	m	no	c.1249delA	r.(?)	p.Ile417Serfs*56	FS	1	nd
39	21	m	yes	c.1259_1260insT	r.(?)	p.Ser421fs*8	FS	1	nd
Tsipi et al. 112 [33]	43	m	yes	c.1275G>A	r.(?)	p.Trp425*	NS	1	nd
81	56	m	no	c.1315C>T	r.(?)	p.Leu439Phe	MS	1	nd
240	40	f	yes	c.1318C>T	r.1318c>u	p.Arg440*	NS	1	nd
60	66	m	no	c.1318C>T	r.1318c>u	p.Arg440*	NS	1	nd
237	4	m	yes	c.1381C>T	r.1381c>u	p.Arg461*	NS	1	nd
206	4	m	yes	c.1381C>T	r.1381c>u	p.Arg461*	NS	1	nd
233	12	m	no	c.1392+1G>A	r.(?)	p.(?)	SS	1	nd
178	31	m	no	c.1392+1G>T	r.(?)	p.Ser421_Pro464del	SS	1	nd
58	16	f	no	c.1393-3_1393-2delTA	r.1393_1527del	p.Ser465_Cys509del	SS	1	nd
124	53	f	no	c.1393-?_2325+?del	r.(?)	p.Ser465_Glu775del	LD	1	CSRD
17	40	f	no	c.1399_1400insA	r.(?)	p.Thr467Asnfs*3	FS	1	nd
200	9	f	yes	c.1453G>T	r.(?)	p.Glu485*	NS	1	nd
Bonatti et al. 22 [37]	na	m	yes	c.1462delA	r.(?)	p.Ser488Alafs*10	FS	1	nd
91	49	f	no	c.1466A>G	r.1466_1527del	p.Tyr489*	SS	1	nd
96	13	f	yes	c.1466A>G	r.1466_1527del	p.Tyr489*	SS	1	nd
Tsipi et al. 87 [33]	8	m	yes	c.1466A>G	r.1466_1527del	p.Tyr489*	SS	1	nd
126	43	f	no	c.1466A>G	r.1466_1527del	p.Tyr489*	SS	1	nd
172	46	m	no	c.1466A>G	r.1466_1527del	p.Tyr489*	SS	1	nd
221	14	f	no	c.1466A>G	r.1466_1527del	p.Tyr489*	SS	1	nd
Terzi et al. 679 [40]	na	na	yes	c.1525_1526insT	r.(?)	p.Cys509Leufs*2	FS	1	nd
104	61	m	no	c.1527+5G>A	r.1393_1527del	p.Ser465_Cys509del45	SS	1	nd
173	19	f	yes	c.1527+675C>T	r.1527_1528ins116	p.Asp510Argfs17	SS	1	nd
187	39	f	no	c.1541_1542delAG	r.(?)	p.Gln514fs*21	FS	1	nd
157	21	f	no	c.1542delG	r.(?)	p.Lys514fs*10	FS	1	nd
145	5	m	yes	c.1549G>T	r.1549g>u	p.Glu517*	NS	1	nd
163	10	m	yes	c.1603C>T	r.(?)	p.Gln535*	NS	1	nd
93	19	m	yes	c.1658A>G	r.1658a>g	p.His553Arg	MS	1	CSRD
Tsipi et al. 127 [33]	5	na	yes	c.1722-3C>A	r.(?)	p.(?)	SS	1	CSRD
176	18	f	no	c.1722C>G	r.(?)	p.Ser574Arg	MS	1	CSRD
Tsipi et al. 98 [33]	12	m	yes	c.1724C>A	r.(?)	p.Ser575*	NS	1	CSRD
Tsipi et al. 44 [33]	58	na	yes	c.1755_1758delAACT	r.(?)	p.Thr586Valfs*18	FS	1	CSRD
89	3	m	yes	c.1756_1759delACTA	r.(?)	p.Thr586Valfs*18	FS	1	CSRD
169	18	f	yes	c.1756_1759delACTA	r.(?)	p.Thr586Valfs*18	FS	1	CSRD
226	18	f	no	c.1830_1833delTCTT	r.(?)	p.Leu612Lysfs*18	FS	1	CSRD
98	55	m	no	c.1840_1841insTTTT	r.(?)	p.Asn614llefs*2	FS	1	CSRD
14	38	m	no	c.1885G>A	r.1885_1925del	p.Gln616Glyfs*4	SS	1	CSRD
101	4	m	yes	c.1885G>A	r.1885_1925del	p.Gln616Glyfs*4	SS	1	CSRD
220	12	m	no	c.1885G>A	r.1885_1925del	p.Gln616Glyfs*4	SS	1	CSRD
Bonatti et al. 34 [37]	na	m	yes	c.1889T>A	r.(?)	p.Val630Glu	MS	1	CSRD
143	16	f	yes	c.1907_1908delCT	r.1907_1908del	p.Ser636*	FS	1	CSRD
125	36	f	no	c.2019delC	r.(?)	p.Cys673*	FS	1	CSRD
Tsipi et al. 50 [33]	29	na	no	c.2033dupC	r.2033dupc	p.Ile679Aspfs*21	FS	1	CSRD
181	41	f	no	c.2033dupC	r.2033dupc	p.Ile679Aspfs*21	FS	1	CSRD
94	41	m	no	c.2033dupC	r.2033dupc	p.Ile679Aspfs*21	FS	1	CSRD
97	12	f	yes	c.2041C>T	r.2041c>u	p.Arg681*	NS	1	CSRD
254	63	m	no	c.2041C>T	r.2041c>u	p.Arg681*	NS	1	CSRD
203	8	f	yes	c.2041C>T	r.2041c>u	p.Arg681*	NS	1	CSRD
156	72	m	no	c.2041C>T	r.2041c>u	p.Arg681*	NS	1	CSRD
207	13	m	no	c.2041C>T	r.2041c>u	p.Arg681*	NS	1	CSRD
102	29	f	yes	c.2084_2085delTG	r.(?)	p.Trp696Glufs*3	FS	1	CSRD
115	59	m	no	c.2106delT	r.(?)	p.Val703Phefs*45	FS	1	CSRD
161	13	f	yes	c.2131delC	r.(?)	p.Arg711Alafs*37	FS	1	CSRD
245	55	f	no	c.2251+1G>A	r.2002_2251del	p.Asp668Glufs*9	SS	1	CSRD
168	69	m	no	c.2297T>G	r.(?)	p.Ile766Ser	MS	1	CSRD
26	52	m	no	c.2325G>C	r.2252_2325del	p.Arg752Leufs*17	SS	1	CSRD
Tsipi et al. 151 [33]	9	m	yes	c.2326-3T>G	r.(?)	p.(?)	SS	1	CSRD
22	40	m	no	c.2329T>A	r.(?)	p.Trp777Arg	MS	1	CSRD
236	12	m	no	c.2352G>C	r.(?)	p.Trp784Cys	MS	1	CSRD
16	37	f	no	c.2356delC	r.(?)	p.Gln786fs*5	FS	1	CSRD
100	32	f	no	c.2409+1insCCC	r.2326_2409del	p.Ala776_Gln803del	SS	1	CSRD
128	59	m	no	c.2409+1G>T	r.2326_2409del	p.Ala776_Gln803del	SS	1	CSRD
235	12	f	no	c.2409+1G>T	r.2326_2409del	p.Ala776_Gln803del	SS	1	CSRD
154	29	f	yes	c.2410-18C>G	r.2409_2410ins2410-17_2410-1	p.Gln803fs*23	SS	1	CSRD
Calì et al. 25 [38]	7	m	yes	c.2446C>T	r.(?)	p.Arg816*	NS	1	CSRD
55	50	f	no	c.2492_2493dupCA	r.2492_2493dupca	p.Asp832Glnfs*10	FS	1	CSRD
227	13	f	no	c.2537_2538ins TCAACATGACTGGCTTCCTTTGTGC	r.2537_2538ins25	p.Leu847Glnfs*26	FS	1	CSRD
182	44	f	no	c.2540T>C	r.2540u>c	p.Leu847Pro	MS	1	CSRD
73	21	f	yes	c.2540T>C	r.2540u>c	p.Leu847Pro	MS	1	CSRD
90	18	m	no	c.2571delTinsAG	r.(?)	p.Ser858Leufs*7	FS	1	CSRD
231	18	m	no	c.2669delC	r.2669del	p.Pro890Leufs*12	FS	1	CSRD
5	21	m	yes	c.2674_2674delA	r.2674_2674del	p.Ser892Alafs*10	FS	1	CSRD
149	18	m	no	c.2693T>C	r.(?)	p.Leu898Pro	MS	1	CSRD
67	51	f	no	c.2730_2731insAAGTGGGA	r.(?)	p.Leu911Lysfs*16	FS	1	nd
122	45	f	no	c.2850+1G>T	r.2618_2850del	p.Lys874Phefs*4	SS	1	CSRD
15	7	m	yes	c.2850G>A	r.(?)	p.Lys874Phefs*4	SS	1	CSRD
Tsipi et al. 124 [33]	11	na	yes	c.2858T>A	r.(?)	p.Leu953*	NS	2	nd
246	45	f	no	c.2915T>C	r.2915u>c	p.Leu972Pro	MS	2	nd
158	12	m	yes	c.2990+1G>T	r.(?)	p.(?)	SS	2	nd
150	38	m	yes	c.2990+5G>C	r.2851_2990del	p.Leu952Cysfs*22	SS	2	nd
142	35	f	no	c.2991-2A>G	r.(?)	p.Tyr998_Arg1038del	SS	2	nd
190	42	f	no	c.3047_3048delGT	r.(?)	p.Cys1016Serfs*4	FS	2	nd
251	25	m	no	c.3062_3063insGT	r.(?)	p.Met1022*	FS	2	nd
Tsipi et al. 170 [33]	11	na	no	c.3076A>T	r.(?)	p.Arg1026*	NS	2	nd
223	14	f	no	c.3104T>G	r.3104u>g	p.Met1035Arg	MS	2	nd
Trevisson et al. I [35]	41	f	no	c.3112A>G	r.(?)	p.Arg1038Gly	MS	2	nd
Trevisson et al. II [35]	30	f	no	c.3112A>G	r.(?)	p.Arg1038Gly	MS	2	nd
10	14	m	yes	c.3168_3169insTA	r.(?)	p.Ala1057*	FS	2	nd
29	8	f	yes	c.3198-2A>G	r.3198_3199del	p.Asp1067fs*20	SS	2	nd
Lin et al. 1 [41]	53	f	no	c.3236_3240dupTTCTA	r.(?)	p.Ala1081Phefs*2	FS	2	nd
134	17	f	no	c.3311T>G	r.3311_3314del	p.Leu1104Hisfs*7	SS	2	TBD
164	28	f	no	c.3456_3459delACTC	r.3456_3459del	p.Leu1153Metfs*4	FS	2	TBD
99	22	f	yes	c.3496+1G>A	r.(?)	p.Tyr1106Leufs*28	SS	2	TBD
217	12	f	no	c.3520C>T	r.(?)	p.Gln1174*	NS	2	TBD
38	49	m	no	c.3574G>T	r.(?)	p.Glu1192*	NS	2	TBD
138	33	f	no	c.3610C>G	r.(?)	p.Arg1204Gly	MS	2	GRD
18	46	f	no	c.3708+1G>C	r.3497_3708del	p.Leu1167*	SS	2	TBD
Ulusal et al. 7 [34]	57	f	yes	c.3709-2A>G	r.3709_3718del	p.Asp1237Leufs*26	SS	2	GRD
13	31	f	yes	c.3721C>T	r.(?)	p.Arg1241*	NS	2	GRD
241	42	f	yes	c.3721C>T	r.(?)	p.Arg1241*	NS	2	GRD
110	55	m	no	c.3739_3742delTGTT	r.(?)	p.Phe1247Ilefs*18	FS	2	GRD
42	16	m	yes	c.3826C>T	r.3826c>u	p.Arg1276*	NS	2	GRD
165	35	m	no	c.3826C>T	r.3826c>u	p.Arg1276*	NS	2	GRD
232	19	f	no	c.3826C>T	r.3826c>u	p.Arg1276*	NS	2	GRD
243	50	m	yes	c.3826C>T	r.3826c>u	p.Arg1276*	NS	2	GRD
191	43	f	yes	c.3826_3828delCGAinsTACT	r.3826_3828delcgainsuacu	p.Arg1276Tyrfs*8	FS	2	GRD
209	11	m	no	c.3827G>A	r.3827g>a	p.Arg1276Gln	MS	2	GRD
54	61	m	yes	c.3827G>C	r.(?)	p.Arg1276Pro	MS	2	GRD
49	22	m	yes	c.3844delA	r.(?)	p.Ser1282Valfs*3	FS	2	GRD
155	25	m	yes	c.3847delA	r.(?)	p.Ile1284*	FS	2	GRD
88	45	f	no	c.3859delT	r.(?)	p.Phe1287Serfs*22	FS	2	GRD
Tsipi et al. 115 [33]	6	na	yes	c.3870_3871insTAG	r.(?)	p.Val1291*	NS	2	GRD
50	46	m	no	c.3888T>G	r.(?)	p.Tyr1296*	NS	2	GRD
70	6	f	yes	c.3916C>T	r.3916c>u	p.Arg1306*	NS	2	GRD
36	59	m	no	c.3916C>T	r.3916c>u	p.Arg1306*	NS	2	GRD
167	64	f	no	c.3941G>A	r.(?)	p.Trp1314*	NS	2	GRD
192	26	m	no	c.3974+1G>A	r.(?)	p.(?)	SS	2	GRD
83	40	f	no	c.3974+1G>T	r.3871_3974del	p.Tyr1292Argfs*8	SS	2	GRD
151	40	m	no	c.3974+2T>G	r.(?)	p.(?)	SS	2	GRD
255	44	f	no	c.3975-2A>G	r.3975_3979delguuag	p.Leu1326Thrfs*6	SS	2	GRD
117	16	m	yes	c.3975-?_4110+?	r.(?)	p.Leu1326Trpfs*14	LD	2	GRD
219	11	m	no	c.3983_3986delCATC	r.3983_3986del	p.Pro1328Glnfs*14	FS	2	GRD
77	67	f	yes	c.3989_3992delAGAG	r.(?)	p.Glu1330Alafs*12	FS	2	GRD
132	46	f	no	c.4054delA	r.(?)	p.Ser1352Valfs*3	FS	2	GRD
9	38	m	yes	c.4084C>T	r.(?)	p.Arg1362*	NS	2	GRD
21	10	f	yes	c.4084C>T	r.(?)	p.Arg1362*	NS	2	GRD
116	10	f	yes	c.4084C>T	r.(?)	p.Arg1362*	NS	2	GRD
Tsipi et al. 106 [33]	6	f	yes	c.4084C>T	r.(?)	p.Arg1362*	NS	2	GRD
113	45	m	no	c.4084C>T	r.(?)	p.Arg1362*	NS	2	GRD
140	30	f	no	c.4084C>T	r.(?)	p.Arg1362*	NS	2	GRD
179	5	m	yes	c.4110+1G>C	r.(?)	p.(?)	SS	2	GRD
Tsipi et al. 96 [33]	54	na	no	c.4134C>T	r.(?)	p.Gln1378*	NS	2	GRD
189	31	f	no	c.4154delG	r.4154del	p.Gly1385Glufs*22	FS	2	GRD
Tsipi et al. 161 [33]	11	na	yes	c.4174G>C	r.(?)	p.Arg1391Thr	MS	2	GRD
75	25	f	yes	c.4267A>G	r.4267a>g	p.Lys1423Glu	MS	2	GRD
Tsipi et al. 177 [33]	6	m	yes	c.4269+1delG	r.(?)	p.(?)	SS	2	GRD
Bonatti et al. 62 [37]	na	m	yes	c.4269+1G>C	r.4111_4269del	p.Val1371_Lys1423del	SS	2	GRD
137	47	m	no	c.4353delT	r.(?)	p.Phe1451Leufs*11	FS	2	GRD
92	40	f	no	c.4402_4406delAGTGA	r.4402_4406del	p.Ser1468Cysfs*5	FS	2	GRD
72	5	m	yes	c.4435A>G	r.4368_4435del	p.Phe1457*	SS	2	GRD
Tsipi et al. 95 [33]	6	na	yes	c.4474G>A	r.(?)	p.Trp1491*	NS	2	GRD
147	46	m	no	c.4515-1G>A	r.(?)	p.(?)	SS	2	GRD
196	56	m	yes	c.4537C>T	r.4537c>u	p.Arg1513*	NS	2	GRD
211	10	m	no	c.4537C>T	r.4537c>u	p.Arg1513*	NS	2	GRD
27	49	f	no	c.4537C>T	r.4537c>u	p.Arg1513*	NS	2	GRD
47	17	m	no	c.4577delG	r.(?)	p.Gly1526Valfs*27	FS	2	GRD
123	34	m	no	c.4606dupA	r.(?)	p.Thr1536Asnfs*7	FS	2	nd
34	48	f	no	c.4630delA	r.(?)	p.Thr1544Profs*9	FS	2	nd
37	14	f	yes	c.4684G>T	r.4684g>u	p.Glu1562*	NS	2	Sec14
146	19	m	yes	c.4817T>A	r.(?)	p.Val1606Asp	MS	2	Sec14
213	14	m	no	c.4867G>C	r.(?)	p.Asp1623His	MS	2	Sec14
Tsipi et al. 68 [33]	11	na	no	c.4959G>A	r.(?)	p.Val1653Ile	MS	2	Sec14
64	18	m	yes	c.4983_4984dupT	r.(?)	p.Asn1662*	FS	2	Sec14
121	23	f	yes	c.5028delG	r.(?)	p.Thr1677Leufs*12	FS	2	Sec14
202	7	f	yes	c.5047A>T	r.(?)	p.Lys1683*	NS	2	Sec14
32	50	f	no	c.5154_5157dupATTC	r.(?)	p.His1720Ilefs*17	FS	2	PH
43	17	f	no	c.5170A>T	r.(?)	p.Lys1724*	NS	2	PH
Tsipi et al. 39 [33]	35	na	no	c.5209T>G	r.(?)	p.Val1736Gly	MS	2	PH
Terzi et al. 320 [40]	na	na	yes	c.5224C>T	r.(?)	p.Gln1742*	NS	2	PH
Terzi et al. 325 [40]	na	na	yes	c.5224C>T	r.(?)	p.Gln1742*	NS	2	PH
25	20	m	no	c.5242C>T	r.(?)	p.Arg1748*	NS	2	PH
152	5	m	yes	c.5276delA	r.5276del	p.Asn1759Metfs*14	FS	2	PH
130	44	f	no	c.5353C>T	r.(?)	p.Gln1785*	NS	2	PH
Tsipi et al. 129 [33]	3	m	yes	c.5382C>T	r.(?)	p.Gln1794*	NS	2	PH
234	13	m	no	c.5425C>T	r.(?)	p.Arg1809Cys	MS	2	PH
214	10	f	no	c.5426G>C	r.(?)	p.Arg1809Pro	MS	2	PH
Tsipi et al.89 [33]	30	m	yes	c.5429G>A	r.(?)	p.Trp1810*	NS	2	PH
41	30	f	yes	c.5471insT	r.(?)	p.Lys1823Asnfs*18	FS	2	nd
111	19	f	no	c.5483A>T	r.(?)	p.Asp1828Val	MS	2	HLR
230	10	m	no	c.5520T>G	r.5520u>g	p.Asn1840Lys	MS	2	HLR
133	10	m	yes	c.5543T>A	r.(?)	p.Leu1848*	NS	2	HLR
Tsipi et al. 46 [33]	28	m	yes	c.5546G>A	r.5206_5546del	p.Gly1737fs*4	SS	2	PH
107	40	f	no	c.5546G>A	r.5206_5546del	p.Gly1737fs*4	SS	2	PH
Tsipi et al. 125 [33]	9	f	yes	c.5546+1G>A	r.5206_5546del	p.Gly1737fs*4	SS	2	PH
62	39	f	no	c.5546+5G>C	r.(?)	p.(?)	SS	2	PH
45	19	f	yes	c.5594T>G	r.5594u>g	p.Leu1865*	NS	2	HLR
48	33	m	no	c.5624C>G	r.(?)	p.Ser1875*	NS	2	HLR
242	24	m	yes	c.5630delT	r.(?)	p.Leu1877Tyrfs*27	FS	2	HLR
222	16	f	no	c.5681T>C	r.5681u>c	p.Leu1894Pro	MS	2	HLR
247	52	f	no	c.5750-177A>C	r.5749_5750ins5750-174_5750-108	p.Ser1917Argfs*25	SS	2	HLR
28	14	f	yes	c.5815delT	r.(?)	p.Cys1939Aspfs*19	FS	3	HLR
205	14	f	yes	c.5839C>T	r.5839c>u	p.Arg1947*	NS	3	HLR
3	42	m	no	c.5839C>T	r.5839c>u	p.Arg1947*	NS	3	HLR
23	12	m	yes	c.5839C>T	r.5839c>u	p.Arg1947*	NS	3	HLR
Tsipi et al. 144 [33]	12	f	yes	c.5839C>T	r.5839c>u	p.Arg1947*	NS	3	HLR
Ulusal et al. 10 [34]	7	m	yes	c.5839C>T	r.5839c>u	p.Arg1947*	NS	3	HLR
248	35	m	no	c.5839C>T	r.5839c>u	p.Arg1947*	NS	3	HLR
159	17	m	yes	c.5851_5852insA	r.(?)	p.Thr1951Asnfs*5	FS	3	HLR
174	49	f	no	c.5890G>T	r.(?)	p.Glu1964*	NS	3	HLR
114	23	m	no	c.5943G>T	r.(?)	p.Gln1891His	MS	3	HLR
33	22	f	no	c.5944-1G>T	r.5944_5950delauuacag	p.Thr1983Lysfs*6	SS	3	HLR
Tsipi et al. 79 [33]	37	na	no	c.6110_6110delT	r.(?)	p.Ile2037Metfs*12	FS	3	HLR
Bonatti et al. 85 [37]	na	f	yes	c.6134delC	r.(?)	p.Thr2045Ilefs*4	FS	3	HLR
78	41	f	no	c.6278delG	r.6278del	p.Gly2093Valfs*36	FS	3	HLR
119	29	m	no	c.6346_6347insA	r.(?)	p.Ser2116Tyrfs*6	FS	3	HLR
4	42	m	no	c.6364+2T>A	r.(?)	p.(?)	SS	3	HLR
201	12	f	yes	c.6365-2A>G	r.6365_6579del	p.Glu2122Glyfs*27	SS	3	HLR
Bonatti et al. 90 [37]	na	f	yes	c.6389_6393delTCAGTinsA	r.(?)	p.Leu2130Hisfs*2	FS	3	HLR
186	47	f	no	c.6477delC	r.6477del	p.Ser2160Valfs*19	FS	3	HLR
11	60	f	no	c.6641+1G>A	r.6580_6641del	p.Ala2194Ilefs*6	SS	3	HLR
239	24	f	yes	c.6641+1G>T	r.6580_6641del	p.Ala2194Ilefs*6	SS	3	HLR
Stella et al.1 [39]	2	f	yes	c.6687_6689delTGT	r.(?)	p.Val2230del	ID	3	HLR
148	55	f	no	c.6688delG	r.(?)	p.Val2230Serfs*14	FS	3	HLR
80	8	f	yes	c.6709C>T	r.6709c>u	p.Arg2237*	NS	3	HLR
86	43	m	no	c.6709C>T	r.6709c>u	p.Arg2237*	NS	3	HLR
108	59	f	no	c.6709C>T	r.6709c>u	p.Arg2237*	NS	3	HLR
249	71	m	no	c.6709C>T	r.6709c>u	p.Arg2237*	NS	3	HLR
204	3	m	yes	c.6709C>T	r.6709c>u	p.Arg2237*	NS	3	HLR
197	5	f	yes	c.6755A>G	r.6642_6756del	p.Phe2215Hisfs*6	SS	3	HLR
216	10	f	no	c.6756+11C>T	r.6642_6756del	p.Phe2215Hisfs*6	SS	3	HLR
210	12	f	no	c.6756+1G>T	r.(?)	p.(?)	SS	3	HLR
198	3	m	yes	c.6770_6771insG	r.6770_6771insg	p.Cys2257Trpfs*6	FS	3	HLR
63	55	f	yes	c.6789_6792delTTAC	r.(?)	p.Tyr2264Glnfs*4	FS	3	HLR-CTD
194	28	m	no	c.6789_6792delTTAC	r.(?)	p.Tyr2264Glnfs*4	FS	3	HLR-CTD
Tsipi et al. 135 [33]	17	f	yes	c.6791_6792insA	r.(?)	p.Tyr2264*	FS	3	HLR-CTD
20	45	f	no	c.6791_6792insA	r.(?)	p.Tyr2264*	FS	3	HLR-CTD
53	29	m	no	c.6792C>A	r.6757_6858del	p.Ala2253_Lys 2286del	SS	3	HLR-CTD
87	54	m	no	c.6792C>A	r.6757_6858del	p.Ala2253_Lys 2286del	SS	3	HLR-CTD
184	9	f	yes	c.6792C>A	r.6757_6858del	p.Ala2253_Lys 2286del	SS	3	HLR-CTD
12	42	m	yes	c.6834delC	r.(?)	p.Thr2279Asnfs*20	FS	3	HLR-CTD
185	4	f	yes	c.6858+3A>T	r.6757_6858del	p.Ala2253_Lys2286del	SS	3	HLR-CTD
66	46	f	no	c.6999+1G>C	r.(?)	p.(?)	SS	3	HLR-CTD
2	25	f	no	c.7096_7101delAACTTT	r.7096_7101del	p.Asn2366_Phe2367del	ID	3	HLR-CTD
82	24	m	yes	c.7096_7101delAACTTT	r.7096_7101del	p.Asn2366_Phe2367del	ID	3	HLR-CTD
69	22	m	no	c.7186_7188delCTA	r.(?)	p.Leu2396del	ID	3	HLR-CTD
52	32	f	yes	c.7192_7193delCT	r.(?)	p.Leu2398Glyfs*2	FS	3	HLR-CTD
Tsipi et al. 128 [33]	5	m	yes	c.7285C>T	r.(?)	p.Arg2429*	NS	3	CTD
252	54	m	no	c.7337C>A	r.(?)	p.Ser2446*	NS	3	CTD
195	3	f	yes	c.7345_7346delAA	r.7345_7346del	p.Asn2449Cysfs*12	FS	3	CTD
225	12	m	no	c.7486C>T	r.7486c>u	p.Arg2496*	NS	3	CTD
Calì et al. 76 [38]	12	f	yes	c.7486C>T	r.7486c>u	p.Arg2496*	NS	3	CTD
218	12	f	no	c.7580_7581dupA	r.(?)	p.Ser2528Ilefs*7	FS	3	CTD
95	26	m	no	c.7619C>G	r.(?)	p.Ser2540*	NS	3	CTD-NLS
Micaglio et al. [36]	23	m	no	c.7686delG	r.(?)	p.Ile2563fs*40	FS	3	CTD
229	11	m	no	c.7703delA	r.(?)	p.Gln2568Argfs*35	FS	3	CTD
19	10	m	yes	c.7720delA	r.(?)	p.Val2575Phefs*28	FS	3	CTD
Tsipi et al. 162 [33]	7	na	yes	c.7725_7726insG	r.(?)	p.Ser2576Valfs*4	FS	3	CTD
40	20	f	no	c.7806+1G>A	r.(?)	p.(?)	SS	3	CTD
56	27	f	no	c.7993C>T	r.7993c>u	p.Gln2665*	NS	3	CTD-SBR
141	20	m	no	c.8111delC	r.(?)	p.Pro2704Glnfs*14	FS	3	CTD-SBR
71	16	m	yes	c.8332G>A	r.8332g>a	p.Val2778Ile	MS	3	CTD

ID code: simple numbers refer to the patients in our cohort; the patients retrieved from the literature are shown using the name of the first author and the code assigned in the original article; na = not available; nd = no domain; f = female; m = male. OPG, optic pathway glioma; CSRD, cysteine/serine-rich domain; TBD, tubulin-binding domain; GRD, GTPase activating protein-related domain; PH, pleckstrin homology-like domain; HLR, HEAT-like repeat regions; Sec-14, Sec14-like domain; CTD, C-terminal domain; NLS, nuclear localisation signal region; SBR, syndecan-binding region; NS, nonsense mutation; FS, frameshift mutation; MS, missense mutation; ID, inframe mutation; SS, splicing mutation; LD, large deletion.

**Table 2:** Mutations in *NF1* gene tertiles and the risk of developing OPG.

Tertile	OPG *n* (%) *n* = 132	Non-OPG *n* (%) *n* = 177	*p*-Value *	OR (95% CI)	*p*-Value **	Total Number
5′ tertile	57 (43.2)	87 (49.2)	0.29	0.28 (0.5–1.23)	0.29	144
Middle tertile	47 (35.6)	57 (32.2)	0.53	1.16 (0.72–1.8)	0.53	104
3′ tertile	28 (21.2)	33 (18.6)	0.57	1.17 (0.66–2.0)	0.57	61

Differences in the frequency of mutations in the different tertiles of the *NF1* gene between the OPG and the non-OPG group. * Chi-squared test; ** logistic regression. OR, odds ratio; CI, confidence interval.

**Table 3:** Mutations in different *NF1* gene regions and the risk of developing OPG.

Regions	OPG *n* (%) *n* = 132	Non-OPG *n* (%) *n* = 177	*p*-Value *	OR (95% CI)	*p*-Value **	Total Number
CSRD	20 (15.2)	30 (16.9)	0.67	0.87 (0.47–1.6)	0.67	50
TBD	1 (0.8)	5 (2.8)	0.24	0.26 (0.3–2.279	0.22	6
GRD	25 (18.9)	25 (14.1)	0.25	1.4 (0.77–2.6)	0.25	50
Sec14-PH	12 (9.1)	11 (6.2)	0.34	1.5 (0.64–3.53)	0.34	23
HLR	25 (18.9)	29 (16.4)	0.55	1.19 (0.66–2.15)	0.56	54
CTD	13 (9.8)	16 (9)	0.80	1.09 (0.5–2.37)	0.80	29
NLS	0	1 (0.6)	1	1 (0.99–1.01)	1	1
SBR	0	2 (1.1)	0.5	1.01 (0.99–1.02)	0.99	2
Others	43 (32.6)	68 (38.4)	0.29	0.77 (0.48–1.24)	0.77	111

Differences in the frequency of mutations in the different domains of the *NF1* gene between the OPG and the non-OPG group. * Chi-squared test; ** logistic regression.

**Table 4:** Distribution of *NF1* mutation types in the OPG and non-OPG group.

Mutation Type	OPG *n* (%) *n*= 132	Non-OPG *n* (%) *n* = 177	*p*-Value *	*p*-Value^§^	OR (95% CI)	*p*-Value **	Total Number
Large deletions	5 (3.8)	3 (1.7)	0.29		2.28 (0.53–9.7)	0.26	8
Frameshift	41 (31.1)	60 (33.9)	0.59		0.87 (0.54–1.42)	0.59	101
Inframe	3 (2.3)	3 (1.7)	0.70		1.34 (0.26–6.7)	0.71	6
Missense	9 (6.8)	24 (13.6)	0.06		0.46 (0.20–10.4)	0.062	33
Nonsense	45 (34.1)	40 (22.6)	0.025	0.15	1.77 (1.07–2.93)	0.26	85
Splicing	29 (22)	47 (26.6)	0.35		0.77 (0.45–1.32)	0.35	76

Differences in the frequency of the different types of the *NF1* gene mutations between the OPG and non-OPG group. * Chi-squared test; ** logistic regression; ^§^ after Bonferroni’s correction.

## Supplementary Materials

The following are available online at https://www.mdpi.com/2072-6694/11/12/1838/s1, Table S1: Mutations in *NF1* gene tertiles and the risk of developing OPG in the combined cohort of our and Anastasaki’s patients; Table S2: Mutations in different *NF1* gene regions and the risk of developing OPG in the combined cohort of our and Anastasaki’s patients.
