# Supplementary material for: Risk of Optic Pathway Glioma in Neurofibromatosis Type 1: No Evidence of Genotype–Phenotype Correlations in a Large Independent Cohort
Source: Cancers (Basel). 2019 Nov 21;11(12):1838. doi: 10.3390/cancers11121838 (PMC6966666; doi:10.3390/cancers11121838)
Supplement: Supplementary file 1 [file cancers-11-01838-s001.pdf]

Supplementary Materials

Risk of Optic Pathway Glioma in Neurofibromatosis Type 1: No Evidence of Genotype–Phenotype Correlations in A Large Independent Cohort

Giulia Melloni, Marica Eoli, Claudia Cesaretti, Donatella Bianchessi, Maria Cristina Ibba, Silvia Esposito, Giulietta Scuvera, Guido Morcaldi, Roberto Micheli, Elena Piozzi, Sabrina Avignone, Luisa Chiapparini, Chiara Pantaleoni, Federica Natacci, Gaetano Finocchiaro and Veronica Saletti

Table S1. Mutations in *NF1* gene tertiles and the risk of developing OPG in the combined cohort of our and Anastasaki's patients.

Tertile	OPG *n* (%) *n*= 259	Non-OPG *n* (%) *n* = 431	*p*-Value *	OR (95% CI)	*p*-Value **	Total Number *n*= 690
5' Tertile	131 (50.6)	187 (43.4)	0.066	1.33 (0.98- 1.81)	0.067	318
Middle tertile	82 (31.7)	154 (35.7)	0.27	0.83 (0.60-1.15)	0.27	236
3' Tertile	46 (17.8)	90 (20.9)	0.31	0.81 (0.55-1.2)	0.53	136

Differences in the frequency of mutations in the different tertiles of the *NF1* gene between the OPG and the non-OPG group. *Chi-squared test; ** logistic regression.

Table S2. Mutations in different *NF1* gene regions and the risk of developing OPG in the combined cohort of our and Anastasaki's patients.

Regions	OPG *n* (%) *n* = 259	Non-OPG *n* (%) *n* = 431	*p*-Value *	OR (95% CI)	*p*-Value **	Total Number *n* = 690
CSRD	49 (18.9)	60 (13.9)	0.81	1.44 (0.95-2.1)	0.82	109
TBD	5 (1.9)	20 (4.6)	0.65	0.40 (0.15 -1)	0.74	25
GRD	41 (15.8)	63 (14.6)	0.66	1.09 (0.71- 1.68)	0.66	104
Sec14-PH	22 (8.5)	24 (5.6)	0.13	1.57 (0.86 -2.86)	0.13	46
HLR	36 (13.9)	78 (18.1)	0.15	0.73 (0.47-1.1)	0.15	114
CTD	25 (9.7)	43(10)	0.89	0.96 (0.57 - 1.6)	0.89	68
NLS	0	1 (0.2)	1	1 (0.99 - 1)	1	1
SBR	0	2 (0.5)	0.53	1 (0.99 - 1.01)	0.99	2
Others	99 (38.2)	169 (39.2)	0.79	0.95 (0.69- 1.31)	0.79	268

Differences in the frequency of mutations in the different domains of the *NF1* gene between the OPG and the non-OPG group. * Chi-squared test; ** logistic regression.

© 2019 by the authors. Licensee MDPI, Basel, Switzerland. This article is an open access article distributed under the terms and conditions of the Creative Commons Attribution (CC BY) license (http://creativecommons.org/licenses/by/4.0/).
